# Risk factors for deep venous thrombosis in women with ovarian cancer

**DOI:** 10.1097/MD.0000000000011009

**Published:** 2018-06-18

**Authors:** Yasuhiko Ebina, Mihoko Uchiyama, Hitomi Imafuku, Kaho Suzuki, Yoshiya Miyahara, Hideto Yamada

**Affiliations:** Department of Obstetrics and Gynecology, Kobe University Graduate School of Medicine, Kobe, Japan.

**Keywords:** clear cell carcinoma, D-dimer, deep venous thrombosis, ovarian cancer, thromboembolism

## Abstract

We aim to clarify the incidence of deep venous thrombosis (DVT) before treatment in women with ovarian cancer and identify risk factors for DVT.

In this prospective study, 110 women underwent venous ultrasonography before cancer treatment and D-dimer levels were measured. We investigated factors predicting DVT by logistic regression.

DVT was detected in 25 of 110 women (22.7%) and pulmonary thromboembolism was coexisted in 2 women (1.8%). A total of 21 women (84.4%) with DVT were asymptomatic. D-dimer levels in women with DVT (median, 10.9; range, <0.5–98.2 μg/mL) were significantly higher than those in women without DVT (2.0; <0.5–60.8 μg/mL; *P* < .01). When 10.9 μg/mL was used as a cutoff value for D-dimer levels to predict DVT, specificity, sensitivity, and positive and negative predictive values were 92.9%, 52.0%, 68.4%, and 86.8%, respectively. The multivariate analysis demonstrated that D-dimer level (odds ratio [OR], 19.7; 95% confidence interval [CI], 5.89–76.76) and clear cell histology (OR, 7.1; 95% CI, 2.12–25.67) were independent factors predicting DVT.

Asymptomatic DVT occurred with great frequency before treatment in patients with ovarian cancer. High D-dimer level and clear cell pathology is associated with a higher DVT risk.

## 1 Introduction

The clinical association between malignant tumor and venous thromboembolism (VTE) was described initially by Trousseau in 1865.^[1]^ From that time on, many studies have shown this link and VTE has been observed in patients with varied cancer type, including ovary, liver, and kidney.^[2]^ Because a large pelvic tumor or massive ascites may compress the intrapelvic veins, women with ovarian cancer are at high risk for VTE. Up to 20% of women with ovarian cancer suffer from VTE.^[3–6]^ Silent subclinical deep venous thrombosis (DVT) may develop and manifest clinically during cancer treatment including surgery. Thromboembolic events, such as pulmonary thromboembolisms (PTE), are the leading cause of death in cancer patients.^[7,8]^ Therefore, accurate diagnosis and appropriate management of VTE before cancer treatment currently is a significant clinically relevant problem.

In diagnostic modalities detecting DVT, contrast venography has long represented the reference standard; however, its use in clinical practice has been limited because of the invasiveness and cost. Venous ultrasonography of the lower extremities is a simple and noninvasive examination, and is performed most commonly in clinical practice. Venous ultrasonography of the lower extremities to diagnose DVT has a sensitivity of 97% and a specificity of 94%.^[9]^ On the other hand, plasma D-dimer is generated via the degradation of fibrin by plasmin, and the level increases with enhanced fibrinolysis secondary to enhanced coagulation. Based on this mechanism, plasma D-dimer levels are used as an index to screen for DVT. A few studies have reported that increased levels of D-dimer are associated with the presence of DVT before treatment for ovarian cancer.^[5,10]^ However, the clinical interpretation of levels of D-dimer and risk of DVT have not been fully elucidated.

We aimed to clarify the incidence of DVT before treatment in women with ovarian cancer, and to identify risk factors for DVT.

## 2 Patients and methods

### 2.1 Patients

This prospective cohort study was approved by the institutional review board of Kobe University Graduate School of Medicine. Between June 2010 and December 2016, 110 consecutive women with pretherapeutic ovarian cancer were enrolled in this study. Informed consent was obtained from all participants. Initial treatment for ovarian cancer was performed at the Department of Obstetrics and Gynecology at Kobe University Hospital. All women underwent plasma D-dimer measurements on the first visits and venous ultrasonography before cancer treatment. No women had known thrombophilia or a previous history of VTE. There were no women who suffered recent infection or trauma.

Ovarian cancer was diagnosed histologically in 96 women, while ovarian borderline malignancy was diagnosed in 14 (serous in 2, mucinous in 12). Clinical characteristics of the 110 women are shown in Table 1. Tumor diameter was evaluated by magnetic resonance imaging. The presence of massive ascites was evaluated using abdominal computed tomography (CT) scan and it was defined as centralization of bowel loops.

**Table 1:**
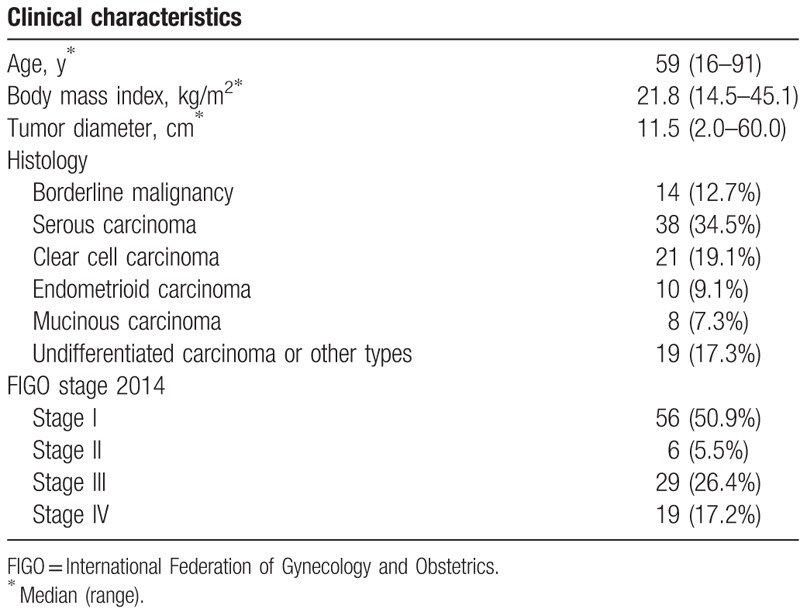
Patient characteristics (N = 110).

### 2.2 Measurement of plasma D-dimer levels

Plasma D-dimer levels were measured at the first visits to the Kobe University Hospital 2 to 5 weeks before initial treatment of ovarian cancer. D-dimer levels were measured using a Sysmex CS-5100 analyzer (Sysmex Corporation, Kobe, Japan) and LPIA-ACE DD-dimer II reagent (LSI Medience Corporation, Tokyo, Japan) sensitized with anti-D-dimer mouse monoclonal antibody to induce a latex coagulation reaction. The cutoff value for plasma D-dimer was 1.0 mg/mL.

### 2.3 Diagnosis of VTE

Venous ultrasonography was performed to detect DVT in all women with pretherapeutic ovarian cancer. Ultrasonography was performed by clinical laboratory technologists using the ultrasound scanner Aplio80 SSA-770A, Aplio500 TUS-A500, or Xario200 TUS-X200 (Toshiba Medical Systems, Tochigi, Japan). Iliac, femoral, greater saphenous, popliteal, peroneal, post-tibial, gastrocnemius, and soleal veins were evaluated bilaterally. Iliac veins were assessed with the patient supine and other veins were assessed with the patient upright. All veins were imaged on transverse and longitudinal views. Venous lumen compressibility then was evaluated by light pressure of the probe. DVT was diagnosed based on the results of venous ultrasonography. Women with DVT systematically underwent enhanced CT of the lungs to find a PTE.

### 2.4 Statistical analysis

Plasma D-dimer levels were compared between women with and without DVT. Next, a receiver operating characteristics (ROC) curve was constructed to determine the cutoff value of D-dimer for the prediction of DVT. Furthermore, univariate and multivariate logistic regression analyses predicting DVT were done using the factors including age (<63 or ≥63 years), body mass index (BMI; <21.7 or ≥21.7 kg/m^2^), pretreatment blood levels of D-dimer (<10.9 or ≥10.9 mg/mL) and CA-125 (<418 or ≥418 IU/mL), tumor diameter (<8 or ≥8 cm), presence of massive ascites, International Federation of Gynecology and Obstetrics (FIGO) stage (stages I/II or III/IV), and histologic type of tumor (borderline or cancer, nonclear cell carcinoma [non-CCC] or CCC). The cutoff values of age, BMI, CA-125, and tumor diameter were also determined using ROC curves. Factors with *P* values less than .05 in the univariate analysis were assessed further by multivariate analysis. Serum concentrations of CA-125 were measured by chemiluminescent enzyme immunoassay with the Lumipulse CA125II (Fujirebio, Tokyo, Japan; normal <35 U/mL).

All statistical analyses were conducted using the statistical package R (ver. 3.2.0, www.r-project.org). The differences between the groups were tested for statistical significance using the Mann–Whitney *U* test and Fisher exact test. The ROC curve methods were conducted to evaluate the diagnostic value for the prediction of DVT. We considered a point at which Youden's J value is maximum (J_max_) as the optimal threshold level. J_max_ was calculated as follows: J_max_ = max_c_ [sensitivity(*c*) + specificity(*c*)–1], when *c* is defined as the threshold level. Statistical significance was determined by *P* < .05.

## 3 Results

### 3.1 Characteristics of the patients with DVT

DVT was found in 25 of 110 women (22.7%) and PTE was coexisted in 2 women (1.8%). Table 2 shows characteristics of the 25 women with DVT. In 25 patients, the site where DVT was located was in the distal part (below the knee level) in 24 patients, in the proximal part in 6, and in both in 5. Thirteen patients (52.0%) showed thrombus only in the soleal vein. Of the 25 women with DVT, 21 (84.4%) were asymptomatic when DVT was found. However, 2 women experienced edema of the lower extremity and 1 had grasp pain of the lower extremity. Only 1 woman with DVT and PTE experienced dyspnea.

**Table 2:**
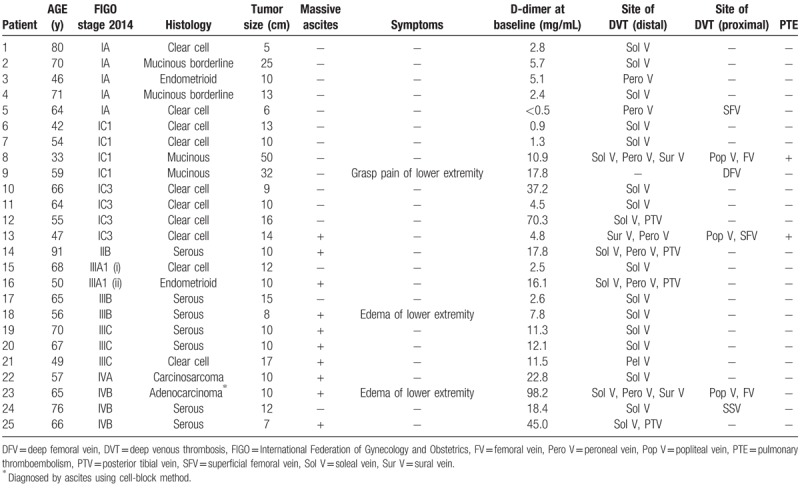
Characteristics of the patients with deep venous thrombosis.

### 3.2 Plasma D-dimer levels and DVT

D-dimer levels were above the reference range (<1.0 μg/mL) in 89 (80.9%) of 110 women with ovarian cancer. Median D-dimer level of 110 women was 2.5 μg/mL (range, <0.5–98.2). The relationship between D-dimer levels and the incidence of DVT is shown in Table 3. DVT was found in 9.5% of the women, even if they had a D-dimer level of <1.0 μg/mL. DVT was noted in 11.9%, 19.2%, and 61.9% of the women with D-dimer levels of 1.0 to 2.9, 3.0 to 10.0, and ≥10.0 μg/mL, respectively (*P* for trend < .01). Increased D-dimer levels were associated with higher incidence of DVT (*P* < .01).

**Table 3:**
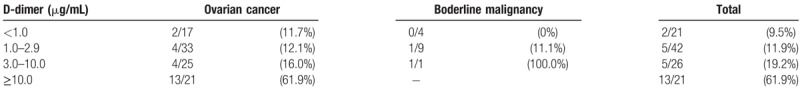
Incidence of thromboembolism for each level of D-dimer.

**Figure 1:**
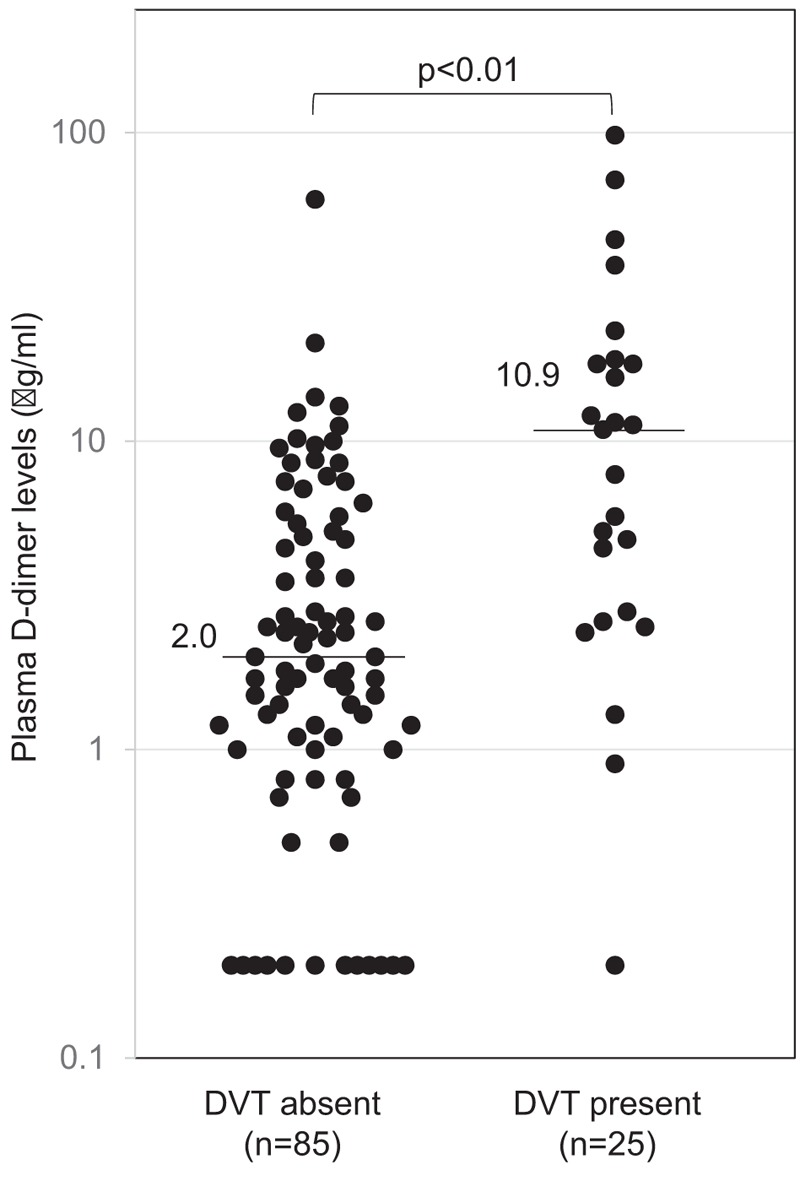
Plasma D-dimer levels before treatment of ovarian cancer in women with and without VTE. Horizontal lines represent median levels. VTE = venous thromboembolism.

**Figure 2:**
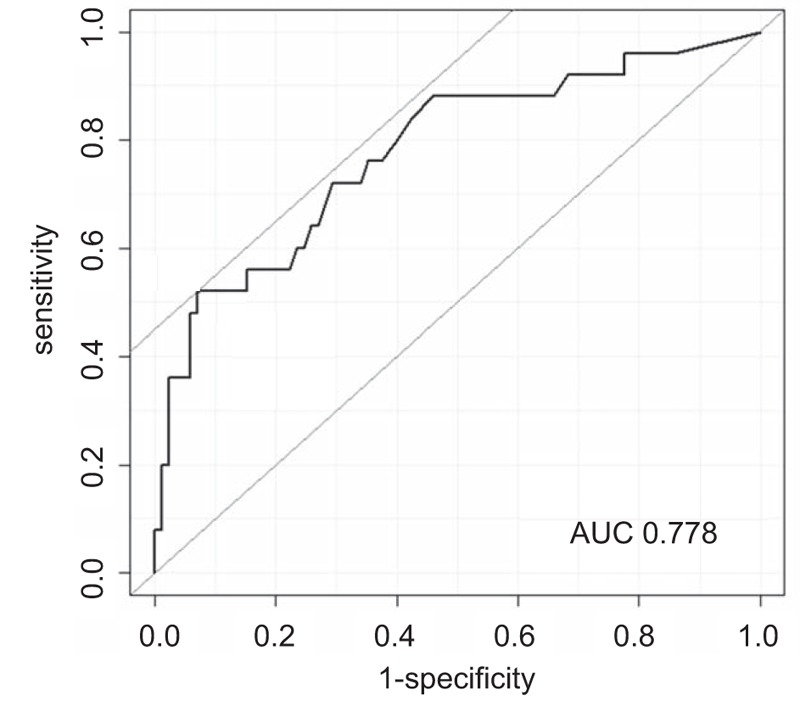
ROC curve for a cutoff value of plasma D-dimer level for the prediction of VTE. ROC = receiver operating characteristics, VTE = venous thromboembolism.

Figure 1 shows D-dimer levels in women with and without DVT. D-dimer levels in women with DVT (median, 10.9; range, <0.5–98.2 μg/mL) were significantly higher than those in women without DVT (2.0; <0.5–60.8 μg/mL; *P* < .01). In the ROC analysis, a relatively high area under the curves (0.778) was obtained when plasma D-dimer levels were used to predict DVT (Fig. 2). Using a cutoff value of 10.9 μg/mL, the optimal results were obtained: 92.9% specificity, 52.0% sensitivity, 68.4% positive predictive value, and 86.8% negative predictive value.

Elevated plasma D-dimer levels were significantly associated with high CA 125 levels (*P* < .01), presence of ascites (*P* < .01), advanced tumor stage (*P* < .01), and malignancy (*P* < .001) but not with age, body mass index, tumor diameter, and clear cell histology (Table 4).

**Table 4:**
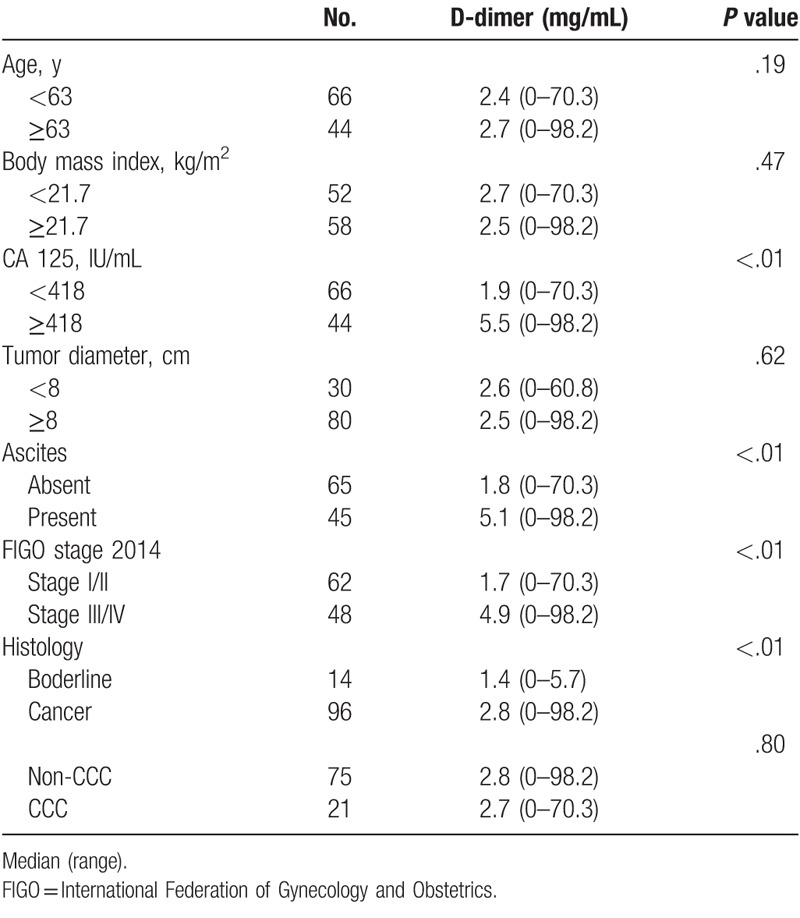
Relationship between clinicopathological parameters and plasma D-dimer level.

### 3.3 Risk factors for DVT

Table 5 shows the relationship between incidence of DVT and characteristics of the women with ovarian cancer. Patients with CCC displayed a higher incidence of DVT compared with those with non-CCC histology (64.5% vs. 16.9%, *P* < .01). Study variables included age, BMI, D-dimer, CA-125, tumor diameter, presence of massive ascites, FIGO stage 2014, and histology. Among these variables, univariate analysis revealed that elevated D-dimer level (≥10.9 μg/mL, *P* < .01) and CCC histology (*P* < .01) were significantly associated with DVT. After multivariate analysis, D-dimer level (odds ratio [OR], 19.7; 95% confidence interval [CI], 5.89–76.76; *P* < .01) and CCC histology (OR, 7.1; 95% CI, 2.12–25.67; *P* < .01) were found to be independent factors predicting DVT.

**Table 5:**
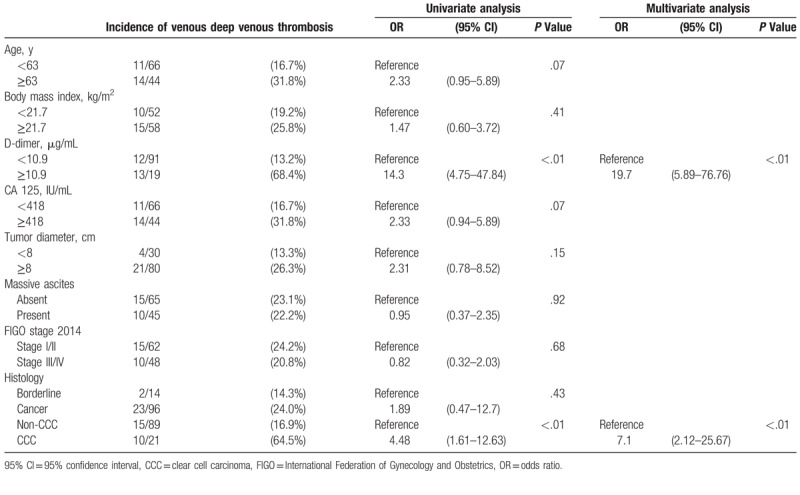
Incidence of deep venous thrombosis by patient characteristics.

## 4 Discussion

In this prospective study, the presence of DVT was assessed using venous ultrasound in 110 women before treatment of ovarian cancer. DVT was detected in 22.7% of the subjects. Several studies have documented preoperative DVT in Japanese patients with ovarian cancer.^[6,10]^ High incidence of DVT (16–25%) was reported. Our data also support that silent or subclinical DVT frequently occurs before treatment. Our study also showed that high D-dimer level and CCC histology were risk factors for DVT.

High plasma D-dimer level is characteristics in common of patients with advanced cancer,^[11]^ and relevant to ovarian tumor burden, CA-125 level, and clinical outcome in patients with ovarian cancer.^[12,13]^ D-dimer levels used to predict DVT have a high sensitivity and negative predictive value of 80% to 100%, but a low specificity and positive predictive value of 20% to 60%.^[9,14]^ Recent studies reported that a suitable cutoff value was 1.5 μg/mL, as this had a 100% sensitivity and 100 negative predictive value.^[6,10]^ In contrast, we demonstrated the optimal cutoff value of 10.9 μg/mL using ROC analysis, with 92.9% specificity, 52.0% sensitivity, and 68.4% positive and 86.8% negative predictive values. If D-dimer was more than 10.0 μg/mL, the frequency of DVT reached more than 60%. However, caution is required when interpreting D-dimer levels. D-dimer levels overlapped between the women with and without DVT (Fig. 1). It was difficult to set a cutoff value from the ROC curve at a glance (Fig. 2). The limitation of this method should be recognized. If cutoff was set at 1.0 μg/mL, 81% of women would become positive. Furthermore, severity of DVT was not associated with D-dimer levels (Table 2). From the perspective of cost-effectiveness, venous ultrasound should be performed when D-dimer is above certain cutoff value.

Among 8 variables predicted as risk factors for DVT, high D-dimer level and CCC histology were significantly associated with DVT. Previous study also demonstrated that CCC histology was a risk factor for preoperative silent VTE^[6]^ or VTE in the postoperative period.^[15,16]^ Some studies have demonstrated that tissue factor (TF) was expressed at higher rates and at higher concentrations in CCC compared with the other types of ovarian cancer.^[17,18]^ TF, which is a membrane glycoprotein, has an extracellular domain that is not in direct contact with the blood flow. Once exposed to the blood flow, the extracellular domain activates coagulation factor VII, which finally product thrombin and fibrin. Recent study demonstrated that TF was involved in the pretreatment development of VTE in ovarian cancer, particularly in CCC.^[19]^

In the guidelines for preventing VTE after general surgery reported in the 9th American College of Chest Physicians evidence-based clinical practice guidelines,^[20]^ women with ovarian cancer are categorized as the highest risk group. These guidelines emphasized a focus on prevention of postoperative VTE and recommended the use of elastic stockings and IPC during and after surgery and anticoagulation therapy postoperatively in women with ovarian cancer. In the 10th guidelines, low-molecular weight heparin over vitamin K antagonist and the direct-acting oral anticoagulants are suggested for initial and long-term treatments of VTE with cancer.^[21]^ These guidelines included no recommendations regarding assessment of silent VTE in pretherapeutic patients. Additionally, risk assessment models including the Caprini and Rogers scores are currently available. These models have demonstrated limited utility in a gynecological oncology patient population because the majority of patients were in the highest risk groups.^[22,23]^

In conclusion, we demonstrated that silent or subclinical DVT occurs frequently before treatment in women with ovarian cancer. High D-dimer level and CCC histology were risk factors for DVT. The findings in our study demonstrated useful information for clinical practice. However, the present study has some limitations. The number of patients was relatively small. The treatments of DVT were different among the women. Further study is necessary to confirm the conclusion of the present study.

## Author contributions

**Data curation:** Mihoko Uchiyama, Hitomi Imafuku, Kaho Suzuki, Yoshiya Miyahara.

**Formal analysis:** Yasuhiko Ebina.

**Funding acquisition:** Hideto Yamada.

**Investigation:** Yasuhiko Ebina, Hitomi Imafuku.

**Methodology:** Yasuhiko Ebina.

**Project administration:** Yasuhiko Ebina.

**Writing – original draft:** Yasuhiko Ebina.

**Writing – review & editing:** Yasuhiko Ebina, Hideto Yamada.
